# Individual variation in the transmission of UVB radiation in the young adult eye

**DOI:** 10.1371/journal.pone.0199940

**Published:** 2018-07-12

**Authors:** Billy R. Hammond, Lisa Renzi-Hammond

**Affiliations:** 1 Brain and Behavioral Sciences, University of Georgia, Athens, Georgia, United States of America; 2 Institute of Gerontology, University of Georgia, Athens, Georgia, United States of America; University of Rochester Medical Center, UNITED STATES

## Abstract

**Objectives:**

Data obtained mostly from animal models and *ex vivo* samples show that a small portion of ultraviolet light (UV, 300–400 nm) penetrates the cornea and crystalline lens and impinges on the human retina. UV transmission to the retina appears to be unique to the young and some older pseudophakes. In this study, we determine the variation in UV transmission in a relatively homogenous sample of young adults.

**Methods:**

42 subjects were tested (*M* = 19 ± 1.3 years). Absolute thresholds to UV radiation were collected (λmax = 315 nm, 305–325). Macular pigment optical density (MPOD, measured using heterochromatic flicker photometry) and iris color (using a standardized color scale) were also assessed as potential covariates.

**Results:**

All of the subjects could detect UV radiation at 315 nm but individual variation was large (over a factor of 30). Higher MPOD and darker iridies were not related to UV sensitivity in this young sample. Males, however, were more sensitive to UV than the females (p<0.05).

**Conclusions:**

The large individual differences in UV reaching the retina of younger individuals suggests equally significant vulnerability to the actinic effects of this highly energetic light.

## Introduction

Textbooks on human vision often state that the width of the visible portion of the electromagnetic spectrum ranges from about 400 to 700 nm [1,2] often noting [3] that “we are blind to energy outside of the visible spectrum.” Although likely meant as a simple approximation, we have known for many years [4] that such generalizations are not, strictly speaking, accurate. For example, the original *ex vivo* data from Boettner and Wolter (1962) shows that there is a window of increased ultraviolet transmission through the anterior media that is centered around 320 nm (40 nm full bandpass) such that nearly as much 320 nm radiation reaches the retina as 400 nm light [5]. This window of transmission seems to sharply decline with age [6] until nearly all detectable UVB (280-315nm) and wavelengths up to 320 nm appears to be absorbed by the anterior media past about the age of 30 years. This age-related decline in ultraviolet transmission appears to be relatively sharp [7]. The average transmission of 320 nm radiation by the crystalline lens goes from around 4.57% at age five to less than half of one percent by age 25 [8,9].

Despite such low levels of transmission, the exposure of the retina to UVB is likely clinically significant [9]due to the nature of the phototoxic effect of such short-wave high energy quanta. UVB radiation, for instance, is sufficiently energetic to initiate not only phototoxicity but also photocarcinogenesis due to its ability to effect DNA.

Given that the anterior media is clear enough in children [10] and young adults to transmit this damaging ultraviolet, it follows that this group is particularly vulnerable to its actinic effects. Historically, this also included aphakics [11] or patients with implants without UV absorbing chromophores [12]. Other groups, however, are likely also susceptible due to excessive exposure. This includes professions such as welders, outdoor athletes, and individuals taking drugs that act as photosensitizers [13].

Given the potential risk to the retina, how much UV radiation is transmitted by the anterior media and when exactly this transmission effectively ceases is important to know. Most of what we know in this area comes from *ex vivo* measurements made from extracted media lens, cornea, etc, often from animal models [6]. The *in vivo* data that exists mostly comes from older studies of aphakics [11]. Such data actually show that, in the absence of significant lens absorbance, individuals can respond behaviorally to UVB radiation [14]. This then allows some important questions, that have not been addressed in the near century-long study of UV perception in humans [15], to be posed: for instance, for healthy young adults, how large are individual differences in UV transmission and does ocular pigmentation alter these thresholds (as proposed originally for the macular pigments by Stark, 1987 [16])? In this study, we addressed these questions.

## Materials and methods

### Study population

Forty-two subjects were recruited (*M* = 19 years, *SD* = 1.3). Subjects (17 male, 26 female, 74% white, 26% Asian or African American) were recruited from the student population at the University of Georgia. The task was a simple detection / absolute threshold task, in which visual acuity was not a factor. Inclusion criteria included good overall ocular health self-reported, and no past history of ocular injury or surgery. Participants who habitually used vision correction devices such as glasses or contact lenses were not allowed to wear them during testing. The tenets of the Declaration of Helsinki were adhered to at all times during the course of this study. All participants both verbally consented to participation and provided written informed consent prior to participation. The protocol was approved by the University of Georgia Institutional Review Board.

### Measurements

#### UV measurements

**Fig 1:**
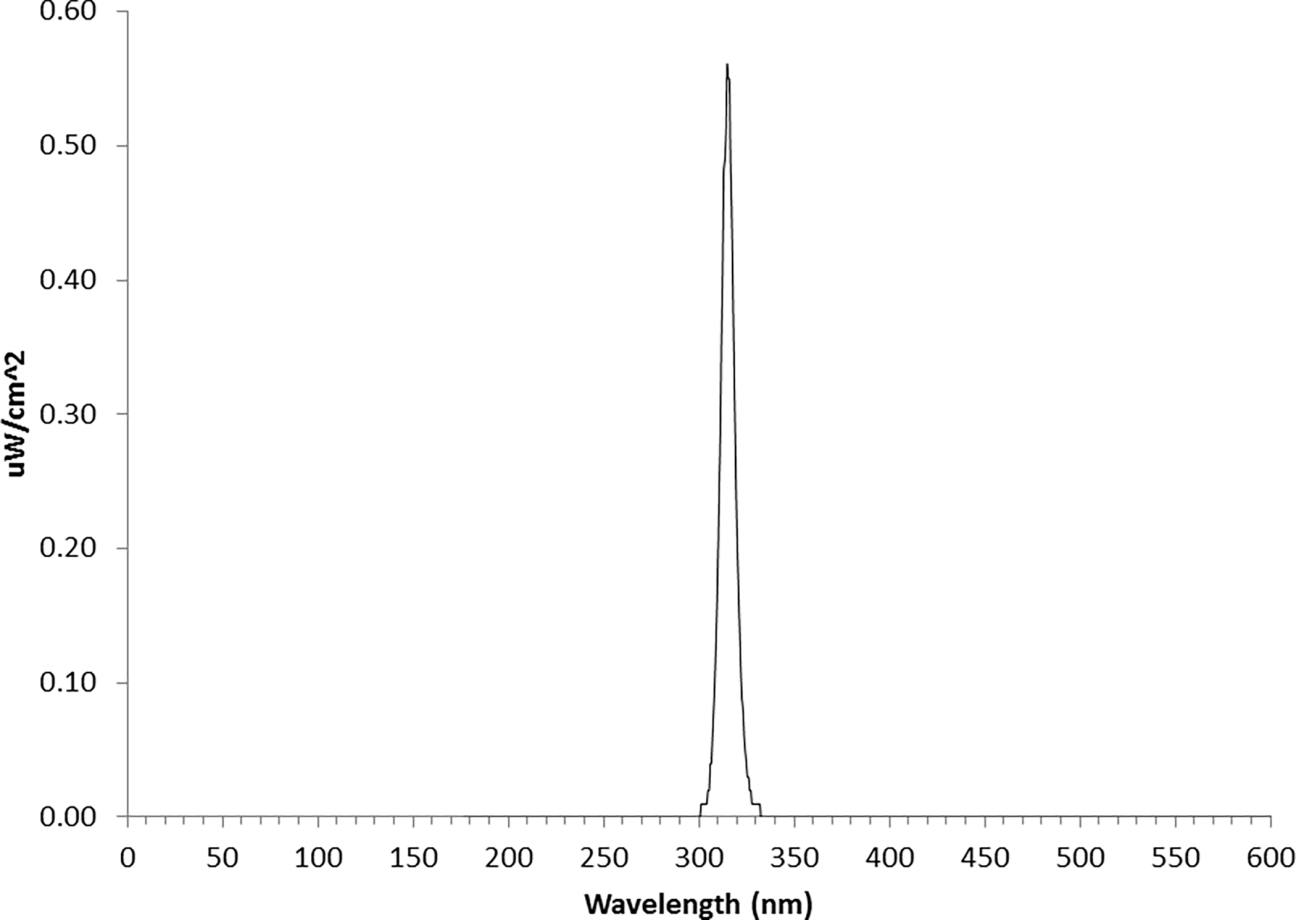
The emittance spectrum of the UV light source. The energy output of the UV light source measured with a calibrated radiometer.

UV radiation was produced using a LED (UVTOP310, QPhotonics, Ann Arbor, MI) with a peak emittance of 313 nm (half bandpass = 10 nm) based on the manufacturer’s specifications. We coupled this LED with a thin-film narrow band interference filter (UV radiation can cause visible fluorescence within the window material of the LED) centered on 310 nm (Pixelteq, Largo, FL). The final spectrum was measured with a spectral radiometer (ILT950, Peabody, MA) to confirm that there was no extraneous visible light and this is shown in Fig 1. This was also checked by simply placing UV-absorbing glass in front of the subjects who could readily detect the UV stimulus without the filter, and confirming that these subjects could not see the stimulus when this material was interposed. The surface of the interference filter (0.35” diameter) served as the viewing surface for the subject and was located 8 inches from the subject’s eye (2.3 degrees visual angle). The intensity of the UV was varied using the LED driver also supplied by QPhotonics. The output of this driver was measured using a calibrated UDT photodiode (S370; Hawthorne, CA) in order to confirm linearity over the measuring range. Subjects were aligned to the same optical axis as the stimulus (viewed with their right eye). We used an adjustable head rest assembly (that included chin and forehead rests) to stabilize alignment and the room was kept dark although subjects were not dark adapted. Exposure of the stimulus was regulated using a mechanical shutter (SC10, Thor Labs, Newton, NJ). The method of constant stimuli was used for the determination of absolute thresholds based on a criterion of 75% detection.

#### Macular pigment and iris color assessment

Macular pigment was determined at peak absorbance (460 nm) using a one-degree measuring stimulus and customized heterochromatic flicker photometry [17]. Measurements were taken on a benchtop device described previously by Wooten et al (1999)[18]. Essentially, this psychophysical technique measures spectral sensitivity using a flicker exchange of wavelengths heavily absorbed by the macular pigments (460 nm) and not absorbed (570 nm) and this is done in the fovea, where the pigments peak, and outside their absorbance at 7-degrees retinal eccentricity. The derivation provides a value for macular pigment optical density that is becoming a recognized standard. Iris color was determined using a set of photographic standards that were presented on a computer monitor based on the system refined by Mackey et al. (2011) [19]. Fifteen samples were shown that varied from light (starting at light gray) to dark (dark brown). One rater was used throughout the study to match the subjects’ iris color to the photographic standards viewed under constant lighting conditions.

## Results

**Fig 2:**
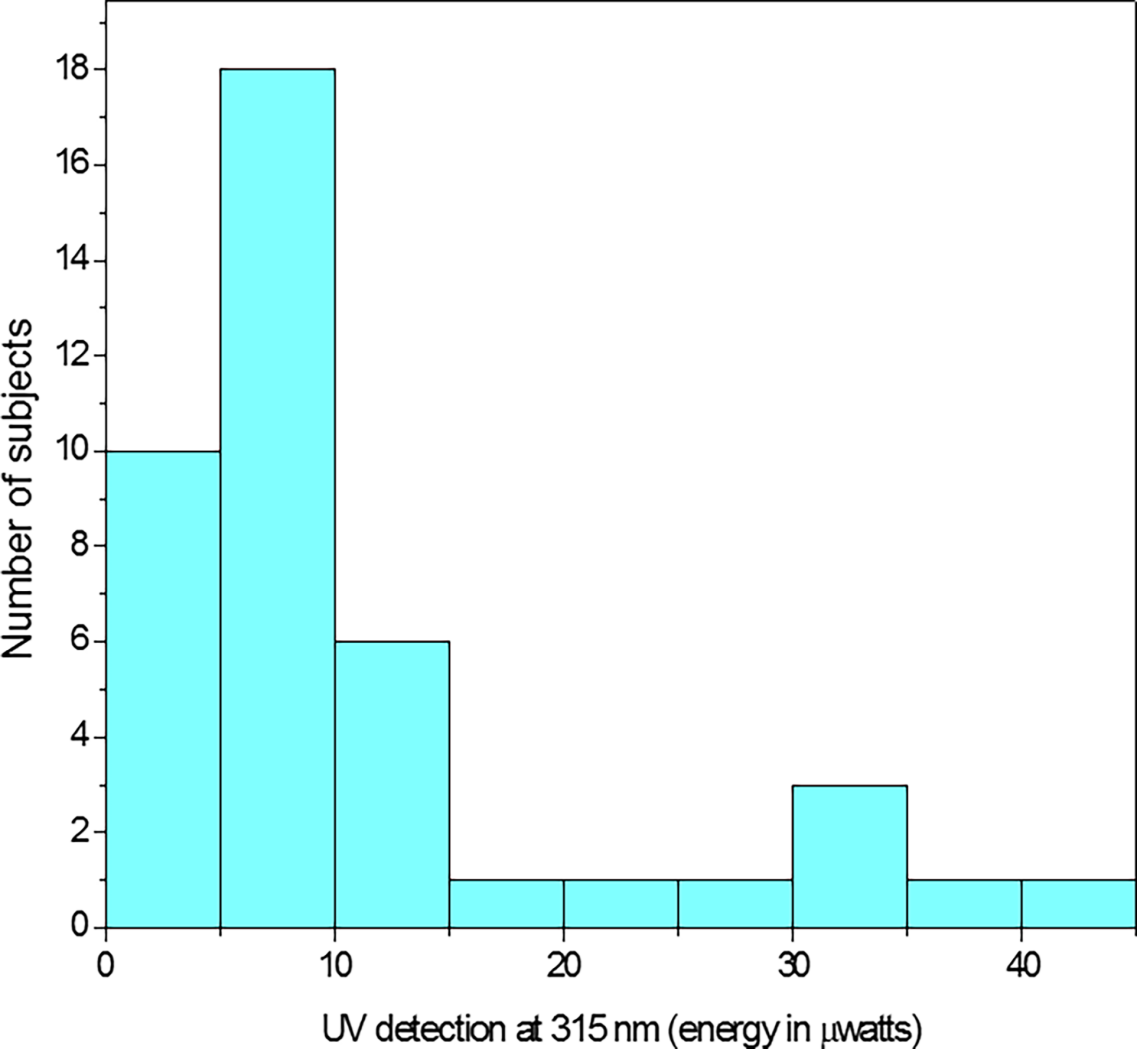
Individual differences in UV thresholds. Individual differences in the amount of energy needed to detect radiation at315 nm.

All of the subjects could detect UV radiation at 315 nm but individual variation was large (over a factor of 30). These results are shown in Fig 2. There was a slight tendency for individuals with higher macular pigment to have reduced sensitivity, but this relation was not statistically significant (r = -0.14, p = 0.37). Iris color also did not appear to correlate with UV sensitivity. For example, light (mean 12.75, sd = 11.95), medium (mean 12.81, sd = 11.85) and dark iris groups (mean 7.82, sd = 7.77) did not differ significantly p = 0.322. The lack of a statistical difference despite large average differences in the dark iris group (e.g., the dark iris group was about 40% lower) was likely due to lack of statistical power linked to the unexpectedly large variation within a category. For example, one 18-year-old white female with light green irides had a threshold at 2.5 microwatts, whereas another 19 year old white female with light green irides had a threshold at 38.5 microwatts. There was a significant mean difference in the UV sensitivity (p<0.05) of the males (n = 16) and females (n = 26) tested in this study. The males had increased sensitivity (mean = 7, sd = 7.97) compared to the females (mean = 13.3, sd = 11.4). This difference was statistically significant p<0.05. There was also a difference based on the habitual use of corrective lenses (not worn during testing). Subjects who habitually wore spectacle or contact lenses (16 females, 9 males) were significantly less sensitive (mean = 1.34, sd = 1.26) to UV compared to those (9 females, 8 males) that did not wear corrective lenses (mean = 0.75, sd = 0.57).

## Discussion

In this study all of the subjects could respond behaviorally to light at 315 nm. Although the fact that young adults could see UV-B was not surprising, it is still common to assume that individuals do not perceive UV-B. Clinical researchers, for instance, have assessed the effects of exposure to UV on numerous disease states (e.g., Maul et al., 2017 [20]) using a double-blind design that is based on subjects presumed inability to perceive UV radiation. Yones et al. (2006), for example, tested the efficacy of UV-A and narrowband UV-B (311–313 nm), in combination with Psoralen, to treat nonsegmental vitiligo [21]: the “double-blind” nature of the study assumed that neither the experimenters nor subjects could see either the UV-A or UV-B radiation. Our results show that such assumptions are not correct.

Many species of birds perceive UV but it is often assumed that the ethologists that study them do not (Eaton et al [22]). For example, Seddon et al. (2009) note [23] that “humans are blind to the near ultraviolet (UV; 300–400 nm) to which many birds are sensitive…” (an oft repeated experimental assumption [24–27]) and that this leads to humans often seeing birds as monochromatic whereas birds can perceive UV-based sex differences in plumage [23]. Nonetheless, Seddon et al. found that, despite appearing monochromatic, “human and avian perceptions of dichromatism were positively correlated.” Such results make sense if the human subjects in their study could also actually respond to UV signals.

The males in our sample were nearly twice as sensitive to UV compared to the females. Also, subjects who did not habitually wear corrective lenses (spectacle and contact lenses) were nearly twice as sensitive to UV as those who do. These specific results, however, should be taken as preliminary since the study sample was too small, and the number in each group was not equal. Nonetheless, links between UVB sensitivity and exposure, refractive condition and sex differences are, at least, feasible. Williams et al. (2017) recently noted [28] that exposure to UVB is related to reduced incidence of myopia possibly due to the effects of the outdoors on growth signals (emmetropization). There are also reports that males and females differ in other aspects of color vision and chromatic filters such as MP. [29]

MP itself has been implicated in absorbing UV. [16] *Ex vivo* studies [30], where MP absorbance is measured with UV-transmitting glass quartz cuvettes, have shown that the macular carotenoids, in their 13-cis form, absorb fairly strongly ~40% of their peak between 300–350 nm. Despite, however, a large range in the MP density of our subjects 0–1.05 OD, we did not find a relationship between UV thresholds at 315 nm and either iris color or macular pigment. This latter finding is inconsistent with the idea that MP is absorbing significant UV in vivo as suggested originally [16] by Stark et al. 1987.

The detection of UVB by these young healthy subjects was fairly easy even at very low light levels (e.g., in blinded phototherapy studies, such as Yones et al.2007, the intensity of the UV-B light is in the milliwatt not microwatt range). One possible explanation for this detection is fluorescence of the crystalline lens or fluorophores within the retinal pigment epithelium. The lens seems an unlikely explanation since numerous studies have shown that aphakics can also detect UVB radiation. Zuclich et al. (2005) has also shown [31] that the excitation spectrum of the lens is longer wave (e.g., an exciting wavelength of 350 nm yields a percept near 500 nm) and diffuse producing a veiling illumination across their visual field (as opposed to confined to the target as our subjects described). Lipofuscin fluorescence within the retinal pigment epithelium is more likely but also inconsistent with the perceptual response of our subjects. The fluorescence of lipofuscin, when excited with wavelengths around 315 nm, peaks around 580 nm, [32] which would yield a yellow percept. Our subjects consistently reported that the light appeared a desaturated violet-blue. The most likely mechanism [33,34] is simply photopigment detection in the beta-band. Beta band absorbance of the L-cone opsin (as opposed to fluorescence) is a common explanation for why short-wave light (400–410 nm) appears as a mixture of both red and blue (violet). Douglas and Jeffry 2014 originally argued [35] that UV sensitivity was widespread among mammals, not because of a specific UV receptor (many receptors have secondary absorbance that extends into the UV) but, rather, that lenses commonly transmit at least some UV radiation to which the retina can respond.

What drives these large variations in UVB sensitivity? Recall that these values represent absolute thresholds. Hence, all subjects would have been able to see higher intensities and this highly actinic light would be transmitted to the retina of some dramatically more than others. For instance, two young women in our sample, 18 and 19 years of age, with similarly light irises, differed by a factor of 19 in their UV sensitivity. Such large variation in otherwise homogenous subjects may be due to differences in lens density. Werner 1989 used visual evoked potentials [36] as a criterion response to measure lens optical density across age. He found that lens OD at 400 nm varied by over a log unit across subjects and that this variation was independent of age. Hence, even infants varied by over a log unit at a measuring wavelength of 400 nm. Mainster and Turner (2010) argued [9] that, for individuals under 30 years, UVB posed the highest risk of retinal phototoxicity and choroidal photocarcinogenecity. Our data suggest that this risk is likely quite variable among young healthy adults and may not be significantly moderated by the typical protective mechanisms, iridial and macular pigments.
